# Effect of water temperature on the anesthetic effects of alfaxalone in carp (Cyprinus carpio)

**DOI:** 10.5455/javar.2024.k743

**Published:** 2024-03-12

**Authors:** Kenichi Maeda, Daiki Hotta, Takuma Matsui, Satomi Iwai, Shozo Okanao

**Affiliations:** Kitasato University School of Veterinary Medicine, Towada, Japan

**Keywords:** Alfaxalone, anesthesia, carp, water temperature

## Abstract

**Objective:**

To evaluate the effect of water temperature on intramuscular injected alfaxalone anesthesia in carp (*Cyprinus carpio*).

**Materials and Methods:**

Six healthy adult carp (*C. carpio*) were intramuscularly injected with alfaxalone (2.5, 5.0, or 7.5 mg/kg) at normal water temperature (25°C) and at low water temperature (2.5 mg/kg, 15°C). The respiratory rate, heart rate (HR), and anesthesia depth (AD) were evaluated every 5 min for 30 min after administration and every 1 h after 60 min after injection.

**Results:**

The respiratory and HRs did not change significantly upon alfaxalone injection, regardless of dose. However, a dose-dependent increase in AD scores was observed. Furthermore, 2.5 mg/kg alfaxalone injected in 15°C water showed an almost equal anesthetic effect to that of 5.0 mg/kg alfaxalone in 25°C water.

**Conclusion:**

Alfaxalone is readily available, and its anesthetic effect in carp was enhanced by lowering water temperature, illustrating the possibility of intramuscular injection of alfaxalone in fish.

## Introduction

Adequate anesthesia is usually required when handling fish for treatment. However, the knowledge of anesthesia management in fish is limited, especially in perioperative anesthesia. Alfaxalone is an anesthetic agent widely used in both mammals and reptiles and is commonly used to induce anesthesia in veterinary medicine [1–5]. However, a high mortality rate due to an overdose of alfaxalone injection anesthesia was known, and only immersion anesthesia is acceptable [6,7]. Therefore, the dose of alfaxalone should be kept in a low and manageable range, although this may not provide a sufficient anesthetic effect. Given the associated risk, the knowledge about intramuscular alfaxalone injection is lacking. We believe that resolving this issue would allow the use of alfaxalone injection as an additional protocol for anesthesia in fish. Hypothermia affects anesthesia in mammals and is known to reduce anesthetic requirements. Therefore, cooling the body may also have anesthetic effects in fish. Fish are poikilothermic animals and their homeostasis is strongly influenced by environmental temperatures [8]. Thus, the anesthetic effect in fish may be affected by water temperature. Reducing water temperature may potentiate the administration of anesthetic drugs to fish when immersed in cold water.

Although cooling live fish during transport is common, adverse reactions, such as death due to over-cooling and cessation of operculum movement, have been reported [9]. Thus, although a lower water temperature may be life-threatening to fish, it is possible to reduce anesthetic administration to safely sedate fish by appropriately managing water temperature. However, no previous study has investigated the effects of water temperature on anesthesia in fish. The objective of our study was to reveal the effect of water temperature on anesthetic effect in fish via intramuscular alfaxalone injections at normal (25°C) and cold water (15°C) temperatures.

## Materials and Methods

### Ethical approval

In this study, the Institutional Animal Care and Use Committee of Kitasato University Veterinary Medicine reviewed and approved all procedures used (22-074).

### Animals

Adult carp (median weight: 225 gm range: 140–360 gm) were used. The six carps were housed in a 1,500 l fiberglass tank filled with dechlorinated water. The tank was placed indoors and acclimated to the conditions (25°C, pH 6.9–7.6) under a 12-h light and 12-h dark cycle for 8 weeks before the start of the experiment. Carps were fed a commercial diet formulated for carp (Swimmy; Nippon Pet Food Co., Ltd., Tokyo, Japan) at 1% body weight three times per week. All fish were considered healthy based on visual inspection and external parasite evaluation.

### Procedures

Each fish was randomly injected intramuscularly with each dose of alfaxalone (Alfaxan^®^ Multidose; Zoetis Inc., NJ, USA) at 25°C and 2.5 mg/kg alfaxalone at 15°C, respectively, with washout intervals between injections of at least 14 days for crossover studies. All of the agents were injected into the dorsal epaxial muscles (*Macrourus carinatus dorsalis*) caudal to the dorsal fin in the water by gently inducing the carp to the water surface. When cooling the water temperature, the fish were moved into a water tank to immerse in 5°C lower water (20°C). After the fish had acclimatization to the temperature for 120 min, they were moved into 5°C lower water (15°C) again. After the 120 min acclimatization period had lapsed, the fish were injected with 2.5 mg/kg alfaxalone. The heart rate (HR), respiratory rate (RR), and anesthesia depth (AD) were measured before intramuscular administration of alfaxalone. The HR was measured using Doppler flow probes of an ultrasonic diagnostic device (M2424A SONOS-5500; Philips Medical Systems Co., Ltd., Amsterdam, Netherlands). The probe was placed directly over the heart and the blood flow associated with the heartbeat was detected. The HR was measured and recorded. The RR was measured and recorded by determining the number of times that the operculum had opened and closed.

The AD was evaluated by a blinded recorder based on the study by Minter et al. [6]: 1) normal equilibrium and avoiding obstacles; 2) partial loss of equilibrium and partially concerned about obstacles; 3) partial loss of equilibrium and indifference to obstacles; and 4) complete loss of balance and movement. All measurements were performed and recorded before alfaxalone administration, initially every 5 min up to 30 min after administration, and then every 30 min up to 240 min after administration.

### Statistical analysis

The results are expressed as the mean ± standard deviation. RR and HR measurements were compared using Tukey’s test after using an analysis of variance. AD scores were analyzed by one-way analysis of variance followed by the Wilcoxon signed-rank sum test for differences between groups. *p*-values less than 0.05 were considered statistically significant.

## Results

Table 1 shows the change in HR after administration of 2.5, 5.0, or 7.5 mg/kg alfaxalone at normal water temperature (25°C) and 2.5 mg/kg alfaxalone at low water temperature (15°C). At normal and low water temperatures, the HR did not change significantly, regardless of the alfaxalone dose.

Table 2 shows the changes in RR after alfaxalone administration at normal (2.5, 5.0, or 7.5 mg/kg) and low water temperatures (2.5 mg/kg). At normal water temperature, the RR did not change significantly; however, at low water temperature, the RR decreased compared to that at normal water temperature 5–240 min after the injection of alfaxalone.

Table 3 shows the changes in AD. At normal water temperature, the AD increased at all alfaxalone injection doses in a dose-dependent manner. The AD increased to 25 min and 10 min after administration of 2.5 and 5.0 mg/kg alfaxalone, respectively, compared to AD measurements before alfaxalone administration. At low water temperature, an increased AD was observed at 5 min after administration compared to pro-administration, before peaking at 15–60 min after administration, and then decreasing to a value equivalent to that immediately before alfaxalone administration at 240 min post-administration. Furthermore, with regards to AD, 2.5 mg/kg alfaxalone administered at a low water temperature showed a similar anesthetic effect to 5.0 mg/kg alfaxalone and higher than that of 2.5 mg/kg alfaxalone at normal water temperature. The anesthetic effect tended to be enhanced at low water temperatures.

**Table 1.** Changes in HR after administration of 2.5, 5.0, or 7.5 mg/kg alfaxalone at normal water temperature (25°C) and 2.5 mg/kg alfaxalone at low water (15°C) temperature.

Time point	25°C			15°C
	7.5 mg/kg	5.0 mg/kg	2.5 mg/kg	2.5 mg/kg
	HR (bpm)			
0 min	23.0 ± 8.8	18.0 ± 7.6	17.5 ± 4.7	19.0 ± 9.2
5 min	24.3 ± 13.0	21.3 ± 11.1	21.0 ± 3.9	15.5 ± 7.1
10 min	24.1 ± 12.0	23.8 ± 9.6	19.5 ± 6.5	20.7 ± 13.4
15 min	27.3 ± 13.6	24.2 ± 8.5	26.0 ± 12.4	17.0 ± 8.6
20 min	29.5 ± 12.4	31.7 ± 17.9	22.7 ± 8.8	21.2 ± 10.2
25 min	29.3 ± 8.4	30.7 ± 11.3	23.7 ± 10.8	19.7 ± 7.7
30 min	28.8 ± 12.6	24.7 ± 7.1	25.8 ± 10.3	19.3 ± 9.3
60 min	27.1 ± 9.6	24.7 ± 5.2	26.8 ± 12.7	26.2 ± 7.2
120 min	27.5 ± 11.4	23.2 ± 9.3	22.2 ± 6.8	25.5 ± 9.3
180 min	21.3 ± 9.5	23.1 ± 11.7	20.8 ± 8.7	26.3 ± 13.6
240 min	18.0 ± 0.0	21.4 ± 12.3	36.0 ± 18.2	25.6 ± 12.9

**Table 2.** Changes in RR after administration of 2.5, 5.0, or 7.5 mg/kg alfaxalone at normal (25°C) water temperature and 2.5 mg/kg alfaxalone in low (15°C) water temperature.

Time point	25°C			15°C
	7.5 mg/kg	5.0 mg/kg	2.5 mg/kg	2.5 mg/kg
	RR (bpm)			
0 min	71.5 ± 28.6	68.3 ± 24.9	74.0 ± 19.2	39.0 ± 10.6
5 min	74.0 ± 9.0	83.0 ± 19.1	91.0 ± 11.6	55.0 ± 12.2^abc^
10 min	72.0 ± 16.1	77.0 ± 7.0	83.0 ± 14.9	47.0 ± 11.6^abc^
15 min	69.0 ± 13.5	73.0 ± 11.6	75.0 ± 13.0	47.0 ± 12.2^abc^
20 min	68.0 ± 13.5	70.0 ± 6.2	74.0 ± 14.0	49.0 ± 8.8^abc^
25 min	70.0 ± 17.7	66.0 ± 10.0	75.0 ± 14.1	46.0 ± 11.1^ac^
30 min	70.0 ± 20.0	61.0 ± 5.9	73.0 ± 12.2	47.0 ± 12.8^ac^
60 min	67.0 ± 11.6	64.0 ± 13.5	78.0 ± 13.7	43.0 ± 8.8^abc^
120 min	70.0 ± 13.0	64.0 ± 11.8	68.0 ± 9.8	42.0 ± 3.8^abc^
180 min	74.0 ± 11.8	60.0 ± 19.3	62.0 ± 16.3	45.0 ± 10.6^a^
240 min	84.0 ± 16.8	60.0 ± 21.3	90.0 ± 18.7	43.0 ± 4.5^ac^

^a^2.5 mg/kg alfaxalone in low water temperature versus 7.5 mg/kg alfaxalone in normal water temperature.

^b^2.5 mg/kg alfaxalone in low water temperature versus 5 mg/kg alfaxalone in normal water temperature.

^c^2.5 mg/kg alfaxalone in low water temperature versus 2.5 mg/kg alfaxalone in normal water temperature.

**Table 3.** AD after administration of 2.5, 5.0, or 7.5 mg/kg alfaxalone at normal water temperature and 2.5 mg/kg alfaxalone in low water temperature.

Time point	25°C			15°C
	7.5 mg/kg	5.0 mg/kg	2.5 mg/kg	2.5 mg/kg
	AD			
0 min	1.0 ± 0.0	1.0 ± 0.0	1.0 ± 0.0	1.0 ± 0.0
5 min	2.2 ± 0.4^ab^	2.0 ± 0.0^ab^	1.3 ± 0.5	2.0 ± 0.0^ab^
10 min	2.3 ± 0.5^ab^	2.2 ± 0.4^ab^	1.8 ± 0.4 ^a^	2.0 ± 0.0^ab^
15 min	3.5 ± 0.5^abc^	2.5 ± 0.5^ab^	1.8 ± 0.4 ^a^	2.5 ± 0.5^ab^
20 min	3.8 ± 0.4^abc^	2.8 ± 0.4^ab^	1.8 ± 0.4 ^a^	2.5 ± 0.5^ab^
25 min	4.0 ± 0.0^ab^	3.2 ± 0.8^ab^	1.7 ± 0.5 ^a^	3.3 ± 0.8^ab^
30 min	4.0 ± 0.0^ab^	3.2 ± 0.8^ab^	1.7 ± 0.5 ^a^	3.3 ± 0.8^ab^
60 min	3.5 ± 0.8^ab^	3.0 ± 0.9^ab^	1.5 ± 0.5	3.5 ± 0.5^ab^
120 min	2.0 ± 0.0^ab^	1.7 ± 0.5^ab^	1.0 ± 0.0	2.5 ± 0.5^ab^
180 min	1.3 ± 0.5	1.0 ± 0.0	1.0 ± 0.0	1.8 ± 0.4^ab^
240 min	1.0 ± 0.0	1.0 ± 0.0	1.0 ± 0.0	1.0 ± 0.0

^a^aversus 0 min ^b^versus 2.5 mg/kg alfaxalone in normal water temperature.^c^versus 5 mg/kg alfaxalone in normal water temperature.^c^2.5 mg/kg alfaxalone in low water temperature

**Figure 1.**
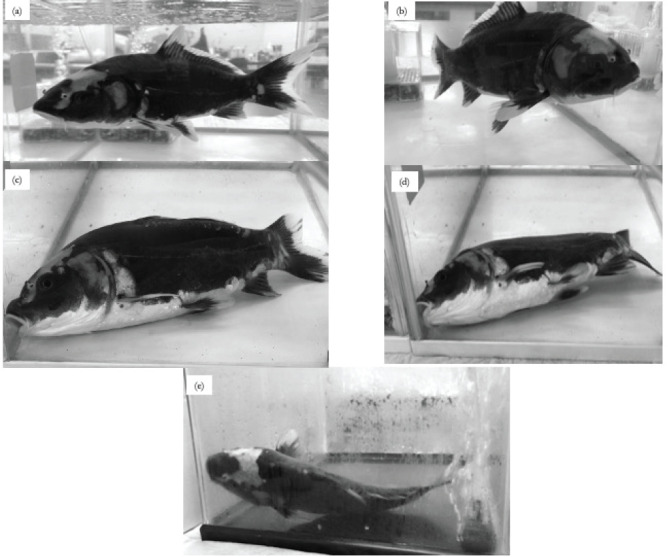
Carp before and after alfaxalone administration images were taken when the highest AD was recorded in each experimental groups (30 min after alfaxalone charrenge). (a) Before alfaxalone, (b) 2.5 mg/kg alfaxalone, (c) 5.0 mg/kg alfaxalone, (d) 7.5 mg/kg alfaxalone, and (e) 2.5 mg/kg alfaxalone (15°C).

Figure 1 shows the rightening reflex before and after alfaxalone administration. At normal water temperature, the carp position was not impacted at 2.5 mg/kg alfaxalone (b). However, the carp were unable to maintain posture at 5.0 and 7.5 mg/kg alfaxalone doses and were immobilized in a lying position (c and d). When 2.5 mg/kg alfaxalone was administered at low water temperature (e), the AD increased compared to that when 2.5 mg/kg alfaxalone was administered at normal water temperature (b), resulting in lateral recumbency, which was similarly observed at 5.0 mg/kg alfaxalone administration in normal water temperature.

## Discussion

In fish, a decrease in environmental temperature directly affects the contractile function of the heart. Moreover, a decreased body temperature with environmental temperature lowers the myocardial cell temperature and suppresses the inflow of calcium ions into cells, serving to prolong the diastole of the heart and increase the stroke volume while decreasing the HR. In general, the Q10 effect, in which the physiological response is changed by a temperature change of 10°C, is recognized in fish as in other animals [9]. In this study, the “low water temperature” was set to 15°C, which was 10°C lower than the “normal water temperature” (25°C); however, there was no significant change in HR due to the Q10 effect. Many fish bodies are thermotolerant to lower body temperatures, as they can be exposed to dramatic changes in temperature due to seasonal changes and migratory waters and depths [10,11]. The carp used in this study were considered to be able to maintain their HR, even with a short-term decrease in water temperature due to heat tolerance, and no significant change was observed during the study period compared to normal water temperature. Moreover, alfaxalone administration did not lead to any significant changes in HR or significant differences between water temperature groups at any time point, corroborating the findings by Bailey et al. [7].

Although there are few reports of alfaxalone intramuscular administration in carp, Bailey et al. [7] reported lethal side effects such as respiratory arrest upon intramuscular administration of 10 mg/kg alfaxalone in carp. In this study, the risk of apnea was predicted, but respiratory depression due to alfaxalone administration was not confirmed.

When fish are exposed to changes in temperature, they may experience thermal adaptation and new functional rates as well as circulatory function, usually accompanied by changes in the respiratory response such as the RR, oxygen consumption rate, and respiratory volume. In this study, the baseline RR tended to be lower due to the decrease in water temperature. Several mechanisms underlie this RR change. A fish’s breathing is affected by the temperature of the water; when a fish is in cold water, its breathing rate slows because the metabolism is reduced with the cold environment and they take in less oxygen and produce less carbon dioxide. Furthermore, the cooling environment itself can have an anesthetic effect by affecting the central nervous system [12]. Furthermore, after administration of alfaxalone, the RR decreases significantly at low water temperatures compared to normal water temperatures. These results indicated that lowering water temperature suppressed respiratory function, which was enhanced by alfaxalone injection. Moreover, given that the dissolved oxygen content increases as the water temperature decreases [13], the RR may have been decreased by increased dissolved oxygen at low water temperature. However, as we did not monitor the changes in dissolved oxygen in this study, the oxygen level in the water is unconfirmed.

In the normal water temperature group, the AD scores increased in a dose-dependent manner upon alfaxalone administration, confirming the anesthetic effect. At 2.5 mg/kg alfaxalone, the AD reached a peak of 1.8 at 10 min after administration. However, the depth of anesthesia was not sufficiently achieved, and immobilization was not obtained at this dose. At 5.0 mg/kg alfaxalone, the AD reached 3.2. Although the AD increased at this dose, complete immobilization was not achieved; however, several experimental, diagnostic, and curative procedures such as biopsy, X-ray, and computed tomography [14–17], might be performed at this dose. At 7.5 mg/kg alfaxalone, the AD reached a maximum level 25 min after administration, indicating that immobilization and a sufficient anesthetic effect were achieved at this injection dose. At low temperatures, after administration of 2.5 mg/kg alfaxalone to carp, the RR was significantly lower than that at normal water temperature; however, the HR was not changed significantly. The AD score peaked at 3.5 at 60 min after administration. A greater anesthetic effect was obtained compared to that at 2.5 mg/kg alfaxalone at normal water temperature. Furthermore, it was found that 2.5 mg/kg alfaxalone administered at low water temperature had the same anesthetic effect as that of 5.0 mg/kg alfaxalone administered at normal water temperature. Drug metabolism is affected by body temperature, and a decrease in body temperature prolongs the onset and elimination of the effect of the drug. In this study, there was no delay in the onset of effects due to the decrease in water temperature. According to a study by Mendonça and Gamperl [18], the resting cardiac output in fish was similar at low and normal water temperatures, and the cardiac function could be maintained.

Furthermore, as described above, the lower water temperature in the present study did not significantly change the HR nor affect blood circulation, which influences drug distribution. Moreover, the recovery of the AD score was delayed at low water temperatures, and it was considered that the metabolism of alfaxalone decreased due to lower water temperature. As poikilothermic animals, the metabolism of fish is depressed in low water temperatures. However, as we only evaluated the HR, the dynamic change in circulation during the experiment is unknown. Therefore, further experiments are necessary to confirm the relationship between lowering water temperature and alfaxalone metabolism in carp.

Cooling fish by lowering the water temperature is considered in transportation and slaughter in fisheries. Hence, we hypothesized that lowering the water temperature would potentiate the anesthetic effects of alfaxalone. In this study, 2.5 mg/kg alfaxalone at low water temperature showed the same anesthetic effect as 5 mg/kg alfaxalone at normal water temperature. Lambooij et al. [12] showed that soaking flounder in ice water and lowering the ambient temperature reduced brain wave output and activity; this can reduce stress and metabolism while maintaining low brain activity and loss of response to painful stimuli. Thus, the stress exerted on the fish body could be sufficiently reduced by decreasing the water temperature at a slow rate. Indeed, adverse reactions, such as death, were observed in any of the carp in the cooling group. In this study, we intramuscularly administered alfaxalone to carp and evaluated the anesthetic effect. To date, there have been few reports of intramuscular administration of alfaxalone in carp, with most studies choosing to use immersion [6]. However, immersion anesthesia requires the use of large amounts of alfaxalone, which requires the dissolution of the anesthetic in water and absorption through the gill, which can be costly and wasteful. With intramuscular administration, it is possible to reliably administer a small dose of anesthetic, which is more economical. When alfaxalone was previously intramuscularly administered to carp at 1, 5, or 10 mg/kg, only 10 mg/kg displayed a sufficient anesthetic effect [7]. However, at a dose of 10 mg/kg, a mortality rate of 33% was recorded, concluding that intramuscular alfaxalone administration in carp was risky and thus not recommended. In this study, intramuscular administration of 7.5 mg/kg alfaxalone achieved anesthetic effects, with a mortality rate of 0%. However, the risks of intramuscular injection of alfaxalone remain and the risk is increased in a dose-dependent manner; therefore, it is important to reduce the dose of alfaxalone. Our findings contribute to the dose-sparing effect of alfaxalone, while maintaining its anesthetic effect, and are expected to improve the possibility to inject alfaxalone intramuscularly for clinical use.

Alfaxalone, like other injectable anesthetics, has no or minimal analgesic effect itself. Several studies have reported on the use of analgesic agents such as buprenorphine in fish [19,20]. In this study, we only investigated the anesthetic effect of intramuscular injection of alfaxalone, not the analgesic effect; therefore, further study is needed to adapt our results relating to analgesic agents to painful procedures or surgery by combining. Furthermore, there are still many uncertain factors in fish anesthesia, including the evaluation of blood gas levels. Future studies are warranted to detail the anesthetic effects of alfaxalone with respect to the changes in water temperature and fish physiology.

## Conclusion

In conclusion, in this study, we report for the first time that lowering water temperature enhances the low-dose alfaxalone anesthetic effect in carp, revealed cooling the water temperature is potent for anesthesia dose-sparing effect in fish. These findings may be generalizable to a variety of fish species by optimizing the degree of cooling water.

## List of Abbreviations

AD, anesthesia depth; °C, degree celsius; HR, heart rate; pH, potential hydrogen; RR, respiratory rate.
